# Histone protein surface accessibility dictates direction of RSC-dependent nucleosome mobilization

**DOI:** 10.1093/nar/gkac790

**Published:** 2022-09-26

**Authors:** Javeed Ahmad Bhat, Angela J Balliano, Jeffrey J Hayes

**Affiliations:** Department of Biochemistry and Biophysics, University of Rochester Medical Center, Rochester, NY 14642, USA; Department of Biochemistry and Biophysics, University of Rochester Medical Center, Rochester, NY 14642, USA; Department of Biochemistry and Biophysics, University of Rochester Medical Center, Rochester, NY 14642, USA

## Abstract

Chromatin remodeling enzymes use energy derived from ATP hydrolysis to mobilize nucleosomes and alter their structure to facilitate DNA access. The Remodels the Structure of Chromatin (RSC) complex has been extensively studied, yet aspects of how this complex functionally interacts with nucleosomes remain unclear. We introduce a steric mapping approach to determine how RSC activity depends on interaction with specific surfaces within the nucleosome. We find that blocking SHL + 4.5/–4.5 via streptavidin binding to the H2A N-terminal tail domains results in inhibition of RSC nucleosome mobilization. However, restriction enzyme assays indicate that remodeling-dependent exposure of an internal DNA site near the nucleosome dyad is not affected. In contrast, occlusion of both protein faces of the nucleosome by streptavidin attachment near the acidic patch completely blocks both remodeling-dependent nucleosome mobilization and internal DNA site exposure. However, we observed partial inhibition when only one protein surface is occluded, consistent with abrogation of one of two productive RSC binding orientations. Our results indicate that nucleosome mobilization requires RSC access to the trailing but not the leading protein surface, and reveals a mechanism by which RSC and related complexes may drive unidirectional movement of nucleosomes to regulate cis-acting DNA sequences *in vivo*.

## INTRODUCTION

The assembly of genomic DNA chromatin, creates an inhibitory environment for trans-acting factors that require access to DNA to carry out critical biological processes such as gene expression (1). Access to DNA is especially restricted with the ∼147 bp core region of nucleosomes, which is in tight association with the core histone octamer, while the linker DNA is generally more accessible to DNA binding factors (2). ATP-dependent chromatin remodeling enzymes help relieve the restrictions imposed by chromatin by using energy derived from ATP-hydrolysis to mobilize nucleosomes to expose cognate sites buried within the nucleosome core region and/or to generate altered nucleosome structures with increased access to the underlying DNA (3–5).

Chromatin remodeling complexes typically contain an ATPase subunit and a number of ancillary subunits that help target and increase remodeling efficiency. Based on homology within the ATPase domain, chromatin remodeling enzymes are grouped into the superfamily 2 (SF2) family of DEAD/H-box helicases and translocases (3–5). Although they do not possess helicase activity, the ATPase domain is able to track along a single strand of DNA in a 3′ – 5′ direction (6–8). ATP-dependent chromatin remodelers can be further grouped into four subfamilies based on homology and domains within the ATPase subunit, defined by archetypal members SWI/SNF, ISWI, INO80/SWR1 and NURD/Mi-2/CHD (4,9). Interestingly, these complexes can exhibit distinct biological activities ranging from altering the structure of chromatin such to enhance DNA accessibility to trans-acting factors and facilitate gene activation, to moving nucleosomes over *cis*-acting elements to promote gene repression (10–13). *In vitro* assays have documented several outcomes of ATP-dependent remodeling complex activities, including the mobilization (translocation) of nucleosomes, generation of structurally altered nucleosome structures, displacement of H2A/H2B dimers, eviction of entire histone octamers, and exchange of wild-type H2A/H2B dimers with variant dimers or visa-versa (4,14).

The RSC (Remodels the Structure of Chromatin) complex is a member of the SWI/SNF (mating-type Switching/Sucrose Non-Fermenting) subfamily of ATP-dependent chromatin remodeling complexes. RSC is a ∼17-protein complex important for activation of a specific subset of genes, including those involved in cell cycle progression, chromosome segregation, stress response pathways, maintenance of cell wall integrity, and can have both repressive and activating effects in transcriptional regulation (15–17). The ATPase-containing subunit in RSC, Sth1, contains a bromodomain, which helps targets the complex to transcriptionally active regions of chromatin by binding to acetylated histone tail domains (18). In contrast to SWI/SNF, RSC is approximately ten times more abundant in yeast (1000–2000/cell) and is essential for mitotic growth (19). RSC depletion in cells results in a global shift of nucleosomes into the nucleosome free region upstream of active genes, suggesting RSC mobilization plays a critical role in promoter structure (11,12,20).

**Figure 1.**
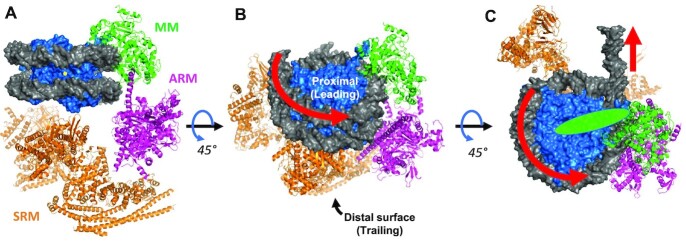
Model of the nucleosome–RSC complex. (**A**). View from ‘back’ of nucleosome, along nucleosome dyad (yellow dot). The motor module (MM) is indicated in green, the actin-related protein module (ARM) in magenta, and the substrate recognition module (SRM) in orange. Note that not shown is the C-terminal portion of the Sth1 subunit containing the SnAc and bromo domains, which extends over the proximal nucleosome face (25). (**B**). Oblique view of the top of the complex. (**C**). View from the top of the complex, looking down the DNA superhelical axis within the nucleosome. Green oval indicates the general predicted location of the SnAc and bromo domains of the C-terminal region of Sth1. Red arrows indicate the direction of DNA movement with respect to the histone octamer, resulting in octamer movement along DNA in direction of the proximal face. The distal (trailing) surface is opposite the leading surface. Image adapted in MacPymol from PDB 6KW4 (23).

*In vitro* experiments show that RSC complexes are able to translocate nucleosomes from centrally located positions to end positions on DNA fragments and can mobilize nucleosomes up to 50 bp past the end of DNA fragments, as shown by photochemical mapping (21,22). Recent cryoEM structures of RSC-nucleosome complexes indicate a multi-modal interaction, with contacts mediated by three modules of the RSC complex (23–25). The Sth1 subunit of the complex contributes to each of the modules and connects them together via flexible regions (23). The RSC motor module is comprised of the ATPase domain of Sth1, and engages the nucleosome DNA at superhelix location (SHL) 2 within the nucleosome (Figure 1). The motor module pumps DNA into the proximal face of the nucleosome, toward the nucleosome dyad, and out of the proximal side of the nucleosome (5) (Figure 1).

The majority of the mass of the RSC complex is comprised of the substrate-recognition module (SRM), which contacts the distal face of the nucleosome, opposite the location of the motor module (23–25). The SRM contains several nucleosome/histone binding domains within multiple polypeptides, including the basic residue-rich C-terminal finger α-helix of Sfh1, which emanates from the ARM module and interacts with the H2A/H2B acidic patch on the distal protein fact of the nucleosome (25), and bromo/BAH domains within Rsc2, Rsc4 and Rsc58, with the tandem bromodomain in Rsc4 exhibiting specificity for H3 K14ac (23–25). Nucleosome contacts are also mediated by the actin-related protein module (ARM), which includes a DNA-binding surface within the HSA domain of Sth1, and actin-related proteins Arp7 and Arp9. The ARM module runs along the side of the nucleosome, connecting the SRM and motor modules. Of note, regions within the C-terminal portion of Sth1 appear to interact with the proximal nucleosome face. These include a C-terminal bromo-domain in Sth1 (25), and the SnAC domain, shown to be a histone anchor important for nucleosome remodeling by the related SWI/SNF complex (26), consistent with a recent structural analysis of the related PBAF-nucleosome complex (27). Thus, both proximal and distal nucleosome faces as well as several DNA surfaces are contacted by the RSC remodeling complex.

A central question is whether the complete array of RSC interactions are essential for nucleosome remodeling. Here we present a method using streptavidin as a steric block to determine whether interaction with specific surfaces of the nucleosome are essential for different remodeling outcomes. We find that nucleosome mobilization activity by RSC is inhibited when a steric block is placed at SHL ±4 via attachment to the N-terminal tail domains of H2A, but that RSC-dependent exposure of an internal DNA site at the nucleosome dyad is unaffected. We also find that interaction of RSC with at least one of the protein faces of the nucleosome is required for RSC remodeling, and that the direction of nucleosome mobilization is dependent upon access to the distal (trailing) protein surface, highlighting a possible mechanism for enforcing unidirectional nucleosome movement *in vivo*.

## MATERIALS AND METHODS

### Histone proteins and nucleosome reconstitution

Yeast RSC was purchased from the Histone Source Protein Expression and Purification Facility at Colorado State University. Recombinant native *Xenopus* core histones and cysteine substitution mutants (either H2A G2C, H2A S128C, H2B S112C) were expressed and purified from *Escherichia coli* BL21 (DE3) cells as described (28,29). In each case a single cysteine was swapped in for either a glycine or serine to minimize the effect of the substitution. H2A/H2B dimers containing cysteine substitutions were incubated with 5 mM maleimide-PEG_2_-biotin at room temperature for 30 min, the modification reaction was stopped with 10 mM dithiolthreitol and samples run on an 18% SDS-PAGE, and stained with Coomassie Brilliant Blue to confirm modification. A 218 bp DNA fragment containing a centrally-positioned 601 nucleosome positioning sequence was generated by radiolabeling the 5′ end of primer TC218F (CGACTGGCACCGGCAAGG) with [α-^32^P]ATP and polynucleotide kinase and performing PCR with TC218R primer (GTAGGGAATACACTACCTG) with p207-601 plasmid as template (Supplementary Table S1). Alternatively, the DNA fragment was labeled with Cy5 near the downstream end by PCR with TC217F and primer 233Cy5HindIIIR (CATc[Cy5]CCTTAAGCTTATGTGATGGACCCTATACG) (see Supplementary Table S1). Nucleosomes were reconstituted with 5 ug of DNA, 5.1 ug of core histones, and were purified over 5–20% sucrose gradients, sedimented at 34 000 rpm for 18 h at 4°C. Purified nucleosomes were stored at 4°C.

### Streptavidin binding to biotin-modified nucleosomes

Biotin modified nucleosomes were incubated with increasing concentrations of streptavidin in TE buffer for 10 min at room temperature before running samples on a 6% native PAGE. A concentration of 10 fmol/μl (0.1 nM) streptavidin was found to be optimal and used in all subsequent experiments.

### Nucleosome remodeling assays

Yeast RSC remodeling assays were performed in 20 μl reactions containing 0.2 nM nucleosomes in a 1× reaction buffer (SWI/SNF: 10 mM Tris, 50 mM NaCl, 5 mM MgCl2, 1 mM DTT and 0.1 mg/ml BSA; RSC: 25 mM Na-HEPES, pH 7.5, 10 mM KCl, 10% glycerol, 2 mM DTT and 0.1 mg/ml BSA) in the presence or absence of 2 mM ATP at 30°C for up to 1 h. Reactions were stopped with 200 ng purified plasmid DNA and run on a 6% native PAGE for 2.5 h at 120 V. Gels were dried and imaged via phosphorimager. All assays were repeated *n* ≥ 3.

### HhaI restriction enzyme accessibility assays

Restriction enzyme accessibility assays were performed during RSC remodeling of nucleosomes in the presence or absence of streptavidin reactions containing 10U HhaI restriction enzyme in 1× RSC remodeling buffer at 30°C. Samples were initially digested with HhaI for 5 min prior to addition of the remodeling enzyme. Timepoints were taken as indicated and added to 6× SDS loading dye solution to stop reactions, then products separated on 6% PAGE containing 0.04% SDS for 2.5 h at 120 V. Gels were dried and exposed to a phosphorscreen overnight and imaged via phosphorimager (Molecular Dynamics). Band densities were quantitated using ImageQuant 5.2 software and the percent of uncut DNA was plotted against HhaI digestion time. The rate of digestion was determined by linear least-squares fits to plots of the ln(%uncut) DNA versus digestion time. Coupled restriction enzyme accessibility/nucleoprotein gel were assays performed a similar manner. Nucleosomes, in the presence or absence of streptavidin, were incubated with 10U HhaI and RSC in the presence of ATP and timepoints were taken at 0, 10, 20, 40 and 60 min during remodeling at 30°C. Reactions were stopped with 200 ng plasmid DNA and 10 mM EDTA and samples were run on a 6% native PAGE for 2.5 h at 120 V. Gels were dried and visualized via phosphorimager. Band densities of the streptavidin-bound species were quantitated as described above. Finally restriction enzyme digestions were done after stopping remodeling by addition of 200 ng plasmid DNA, followed by addition of HhaI and incubation at 37°C.

### Asymmetrically modified nucleosomes

We prepared hexasomes according to published methods (30,31). Hexamers were reconstituted, containing either H2A/H2B dimers or biotin-modified H2A/H2B S112C dimers, then converted to full nucleosomes with modified or native dimers, respectively, to generate asymmetrically modified nucleosomes. Converted nucleosomes were purified by sucrose gradients. Remodeling reactions were carried out as described above, in the presence or absence of streptavidin, and reactions terminated by the addition of 200 ng plasmid DNA. Reactions were digested with 10 u HindIII for 60 min at 37°C then loaded directly onto 5% acrylamide nucleoprotein gels and the wet gels imaged by fluorography (GE Amersham Typhoon). The amount of products before and after HindIII digestion was quantified by densitometric analysis of the fluorographs and the fraction remaining uncut by HindIII calculated and plotted.

## RESULTS

**Figure 2.**
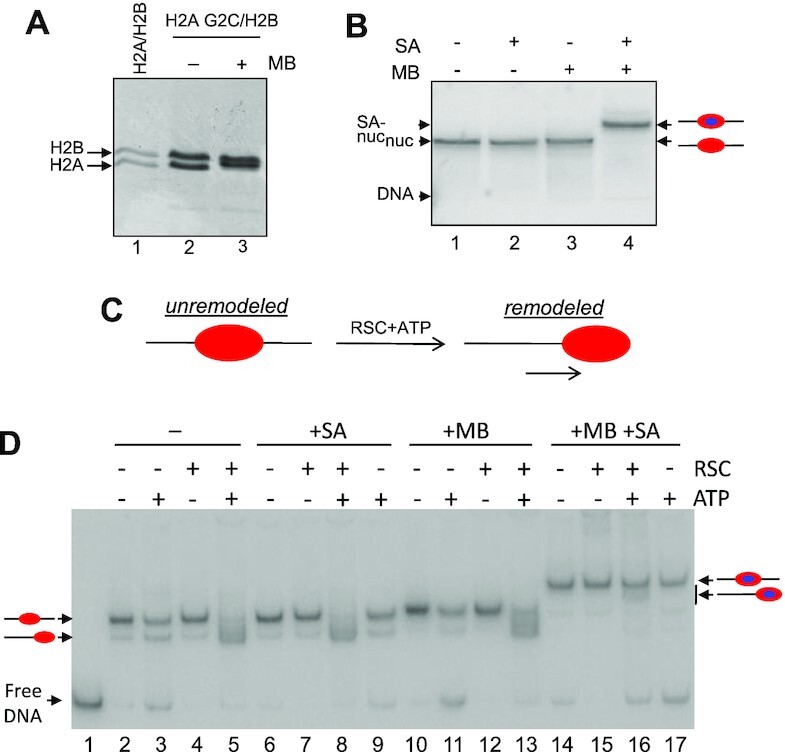
Streptavidin attachment to the H2A N-tail domains inhibits nucleosome mobilization by RSC. (**A**). SDS-PAGE of modified H2A/H2B. Lane 1, WT H2A/H2B; lanes 2 and 3, H2A G2C/H2B before and after MB modification. (**B**). Streptavidin binds to MB-modified H2A G2C nucleosomes. H2A G2C nucleosomes assembled with radiolabeled DNA, containing or lacking MB modification were incubated in the absence or presence of streptavidin (SA), as indicated then run on a 5% acrylamide native gel. (**C**). Schematic of RSC remodeling showing mobilization of nucleosomes from the center to the ends of the 217 bp DNA fragment. (**D**). Streptavidin binding to H2A G2C-MB nucleosomes inhibits nucleosome mobilization by RSC. Nucleosomes containing unmodified (lanes 2–9) or MB modified H2A G2C (lanes 10–17) were incubated either without (lanes 2–5 and 10–13) or with streptavidin (lanes 6–9 and 14–17), then remodeled with ATP and RSC, as indicated above the lanes. The positions of unremodeled (center-positioned) or remodeled (end-positioned) nucleosomes are indicated beside the gel, blue oval indicates streptavidin.

To probe nucleosome surfaces critical for functional RSC interactions, we attached a biotin moiety via modification of a single cysteine residue in either the H2A N-terminal tail domain (H2A G2C) or at a position within H2B (H2B S112C) located near the center of the nucleosome protein surface (Supplementary Figure S1A). Based on crystal structures of the nucleosome and crosslink mapping data (32–34), the cysteines were placed within the H2A G2C N-tails and are ∼20 Å apart, approximately the distance between the two biotin binding sites on one face of the streptavidin tetramer (Supplementary Figure S1A and B) (43), suggesting streptavidin may simultaneously bind to both biotinylated tail domains (see below). H2A G2C nucleosomes modified with maleimido-biotin (MB) have the identical mobility as unmodified nucleosomes on 6% native polyacrylamide gels, suggesting the modification does not alter the efficiency of reconstitution, nucleosome conformation, or positioning (Figure 2A and B). As expected, wild-type, unmodified nucleosomes do not bind streptavidin while biotin-modified H2A G2C nucleosomes show a single, discrete change in mobility, suggesting a single streptavidin binds simultaneously to both H2A N-tail domains in an MB-modification-dependent manner (Figure 2B, see also below).

To determine whether streptavidin binding to H2A G2C-MB nucleosomes affects RSC remodeling we performed nucleosome mobilization assays, wherein migration through the gel increases if nucleosomes are mobilized from their central position on the DNA fragment to an end position (Figure 2C). H2A G2C nucleosomes lacking biotin modification were efficiently remodeled in an ATP-dependent manner, as indicated by the near complete movement of nucleosomes to the end of the DNA fragment, regardless of the absence or presence of streptavidin (Figure 2D, lanes 2–5 and 6–9). Likewise, H2A G2C-MB-modified nucleosomes in the absence of streptavidin were efficiently remodeled by RSC in an ATP-dependent fashion, (Figure 2D, lanes 10–13). In contrast, binding of streptavidin to H2A G2C-MB nucleosomes drastically reduced the ability of RSC to mobilize nucleosomes, as indicated by the lack of a more rapidly migrating species appearing beneath the streptavidin-bound nucleosome band (Figure 2D, compare lanes 14–17). Control experiments show that that streptavidin binding does not mask mobilization on the gel (Supplementary Figure S2). These results indicate that RSC-dependent nucleosome mobilization is impeded when streptavidin is bound to the H2A N-tail domains.

**Figure 3.**
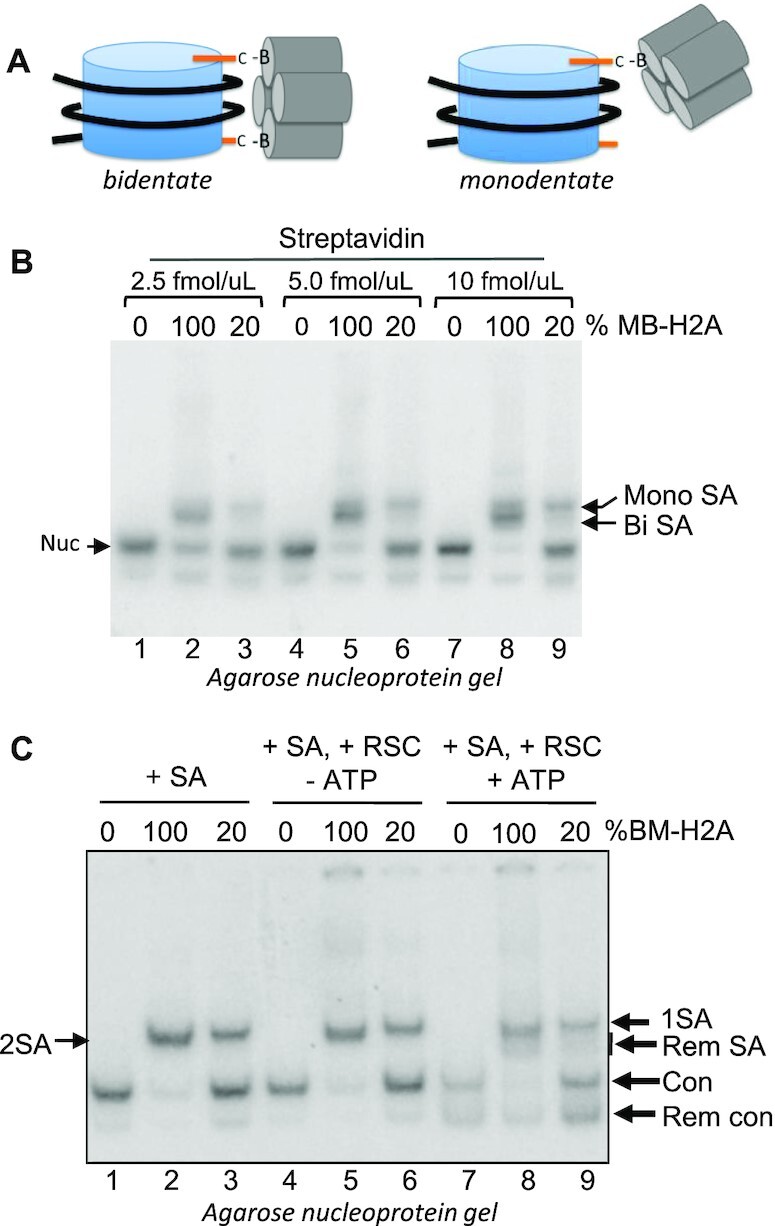
Streptavidin inhibits RSC mobilization of H2A G2C-MB nucleosomes via both monodentate and bidentate binding modes. (**A**). Cartoon of monodentate and bidentate streptavidin binding (also see Supplementary Figure S1). (**B**). Distinguishing monodentate and bidentate streptavidin-bound nucleosomes. Nucleosomes were reconstituted with either 100% MB-modified H2A G2C or a mix of 20% H2A G2C-MB and 80% WT H2A/H2B, as indicated, such that probability of more than one streptavidin in the latter is low (see text). Reconstituted nucleosomes were incubated with increasing amounts of streptavidin (SA) to ensure complete binding and products separated on a 6% native PAGE, the gel dried and visualized by phosphorimager. Bands corresponding to monodentate and bidentate bound streptavidin are indicated. (**C**). Both monodentate and bidentate binding of streptavidin to H2A G2C-MB nucleosomes inhibit nucleosome mobilization by RSC. Nucleosomes lacking biotin or containing primarily one or two biotinylated H2A N-terminal tail domains were incubated with streptavidin (SA), RSC and ATP, as indicated, and products analyzed on a 6% native PAGE as in (B). The location of unremodeled and remodeled species are indicated.

We previously demonstrated that covalently crosslinking the two H2A N-tails together via a small bifunctional crosslinker inhibits nucleosome mobilization, possibly by hindering an internal DNA bulge from passing this location on the nucleosome (28). We therefore asked whether the inhibition of mobilization was due to bidentate streptavidin binding effectively crosslinking the tails together (Figure 2B), or due to simple steric interference. To differentiate between these possibilities, we generated nucleosomes containing only a single biotin-modified H2A N-terminal tail by reconstituting nucleosomes with 80% wild-type (WT) H2A and 20% H2A G2C-MB (Figure 3A). We observed that regardless of the amount of streptavidin added to the reaction, nucleosomes containing two modified H2A N-tails (100% H2AG2C-MB) migrated faster on native gels compared to those containing only a single modified H2A N-tail (20% H2A G2C-MB) (Figure 3B). The faster migrating complex is consistent with a bidentate streptavidin-nucleosome interaction that adopts a more compact structure compared to the monodentate streptavidin-nucleosome complex, expected to have a looser structure and thus migrate more slowly through the gel (Figure 3A). Moreover, we found that monodentate binding of streptavidin to H2A G2C-MB nucleosomes inhibited RSC remodeling activity to an extent similar to bidentate binding (Figure 3C, compare lanes 5 and 8; 6 and 9). These results indicate that the inhibition in RSC mobilization of nucleosomes by streptavidin binding to the H2A N-tails is likely due to steric interference, and indicates RSC requires at least transitory interactions with the nucleosome surface in the vicinity of the H2A N-tails to mobilize nucleosomes.

**Figure 4.**
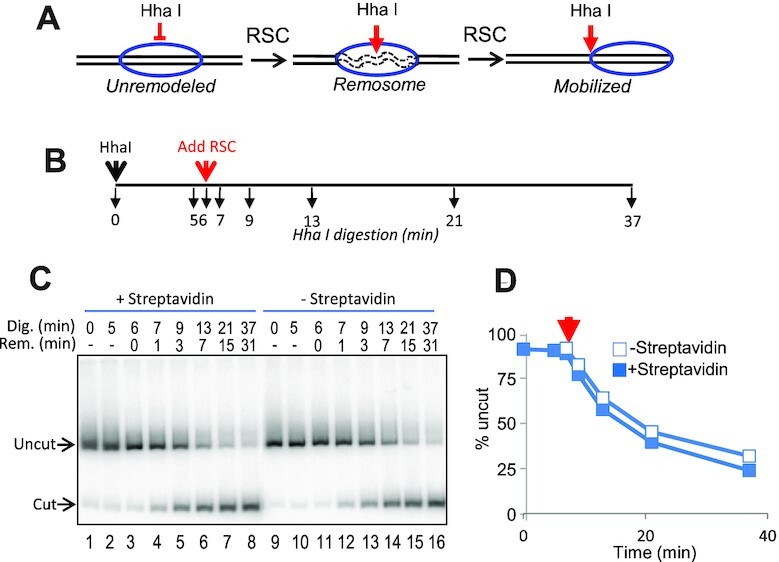
Streptavidin association with H2A N-tail domains does not inhibit RSC remodeling-dependent nucleosome DNA site exposure. (**A**). Detection of RSC remodeling-dependent exposure of the HhaI cognate DNA site at the nucleosome dyad, which can be uncoupled to mobilization (35). (**B**). Experimental timeline. HhaI and RSC were added at *t* = 0 and *t* = 6 min., respectively. Samples of the digest were taken at the times indicated by the black arrows below the line. (**C**, **D**). Products of digestion were run on 5% SDS PAGE gels and quantified by phosphorimagery. (D). Plot of average percent uncut vs time (min). Streptavidin bound and unbound samples are indicated by the open and filled squares, respectively (*n* = 3, avg. SD 4%).

Nucleosome mobilization is just one of several biochemical outcomes of ATP-dependent chromatin remodeling activities. Indeed, ATP-dependent nucleosome remodeling by RSC and the related SWI/SNF complex generates remodeled nucleosomes that exhibit increased accessibility to DNA binding factors prior to nucleosome mobilization (35,36). The increased accessibility can be assessed by restriction enzyme activity (REA) assays that detect internal DNA site exposure via the coupled binding and cleavage of nucleosomal DNA. We therefore determined whether streptavidin association with H2A G2C-MB nucleosomes altered RSC-dependent exposure of a HhaI site in the center of the nucleosome (Figure 4A and B). As expected, little or no digestion by HhaI of unbound or streptavidin-bound nucleosome DNA was observed before addition of RSC, however, upon addition of RSC, there was an immediate increase in HhaI cleavage of nucleosome DNA, with equivalent digestion for both unbound and streptavidin-bound H2AG2C-MB nucleosomes (Figure 4C and D). Therefore, while native gel assays indicate inhibition in RSC-dependent mobilization by streptavidin binding to H2A G2C-MB nucleosomes, this modification did not significantly alter exposure of the internal HhaI site during remodeling.

**Figure 5.**
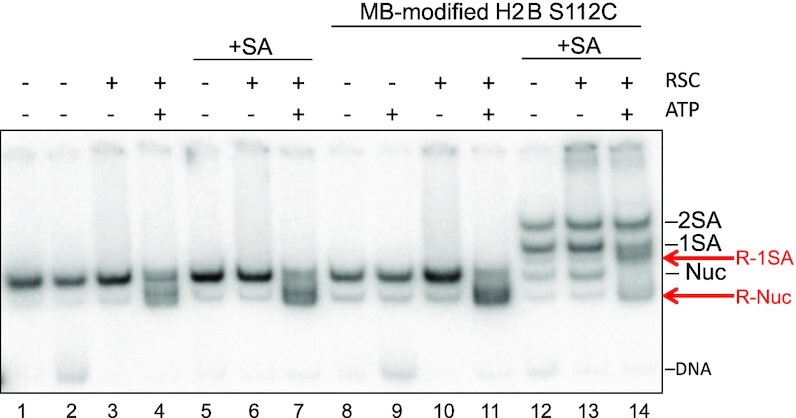
Accessibility of at least one nucleosome protein surface is required for RSC nucleosome mobilization. Nucleosomes were reconstituted with either unmodified (lanes1-7) or MB modified (lanes 8–14) H2B S112C, and incubated in the absence (lanes 1–4 and 8–11) or presence (lanes 5–7 and 12–14) of streptavidin (SA). Samples were incubated with RSC and ATP as indicated and remodeling-dependent nucleosome mobilization assessed on native 6% PAGE. H2B S112C-MB nucleosomes bound by 0, 1 or 2 streptavidins are indicated as Nuc, 1SA or 2SA, respectively (also see Supplementary Figure S2). Remodeled products associated with 1SA and Nuc are indicated by the red arrows and indicated by R-SA1 and R-Nuc, respectively.

We next determined whether access to the flat protein surfaces of the nucleosome are required for RSC remodeling activity. Nucleosomes were generated containing H2B S112C, wherein a cysteine is located near the center of the protein surface of the nucleosome, near the acidic patch (Supplementary Figure S1A and C). Interestingly, streptavidin binding to H2B S112C nucleosomes produced two new species on native gels, consistent with binding of either one or two streptavidins (Figure 5, lane 12, see also Supplementary Figure S3). Of note, this binding profile was not dependent on streptavidin concentration (not shown), indicating that either the dimers were incompletely modified with MB or access to a fraction of the biotin moieties is restricted, perhaps due to nucleosome allostery (37). Nevertheless, the generation of distinctly bound species on native gels allowed us to assess the effect of binding of 0, 1 or 2 streptavidins on remodeling in one reaction (Supplementary Figure S3). When incubated in the presence of RSC and ATP, unmodified H2BS112C nucleosomes in the absence or presence of streptavidin (Figure 5, lanes 1–6) and MB-modified H2B S112C nucleosomes without streptavidin (Figure 5, lanes 8–11) were efficiently mobilized. However, each of the three species generated upon streptavidin binding to H2B S112C-MB nucleosomes were distinctly mobilized by RSC (Figure 5, lanes 12–14). H2B S112C-MB nucleosomes bound by a single streptavidin were mobilized by RSC, but at an apparently reduced rate compared to the remaining unbound nucleosomes, while nucleosomes bound by two streptavidins exhibited little or no mobilization (Figure 5, lanes 13 and 14).

**Figure 6.**
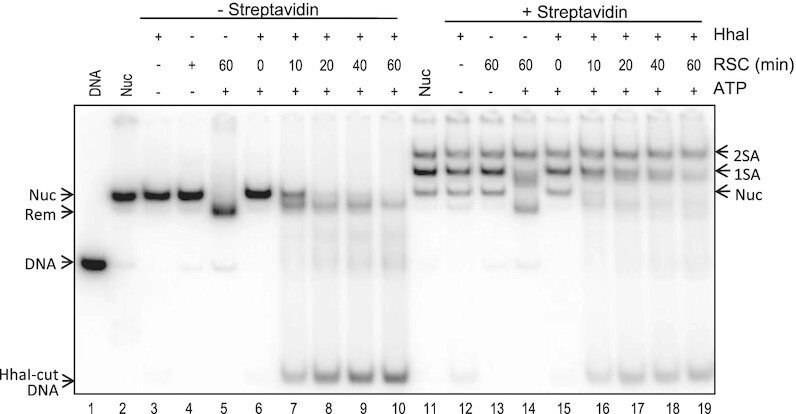
Accessibility of nucleosome protein surfaces indicates two productive RSC-binding orientations. H2B S112C-MB were incubated in the absence (lanes 3–10) or presence of streptavidin (lanes 11–19). Nucleosomes were incubated for 60 min at 30°C with HhaI without RSC (lanes 3 and lane 12, respectively), or without HhaI in the presence of RSC ± ATP (lanes 4 and 5, and lanes 13 and 14, respectively). Additionally, unbound and SA bound nucleosomes were incubated with HhaI, in the presence of RSC and ATP and time points were taken after 0, 10, 20, 40 and 60 min of remodeling at 30°C (lanes 6–10 and lanes 15–19, respectively). Lane 1, free DNA; lanes 2 and 11, unbound and SA bound nucleosomes, respectively. Nuc, 1SA, and 2SA indicate species bound by 0, 1 or 2 streptavidins (see also Supplementary Figure S3).

We then assessed whether RSC remodeling of H2B S112C-MB nucleosomes increased DNA exposure via HhaI restriction enzyme accessibility assays, as described above. While unbound nucleosomes incubated with RSC and ATP were almost completely digested by HhaI, streptavidin-bound H2B S112C-MB nucleosomes were only partially digested by the restriction enzyme, indicating inhibition of RSC-dependent exposure of nucleosome DNA (Supplementary Figure S4). However, since the latter sample contained a mix of H2BS112C nucleosomes bound by 0, 1 or 2 streptavidins, and exhibited multiphasic kinetics, we determined digestion kinetics for individual species by coupling HhaI digestion with analysis on nucleoprotein gels. Control H2B S112C-MB nucleosomes without streptavidin were digested efficiently by HhaI during RSC remodeling, and dissociated on the gel (Figure 6, lanes 6–10). Likewise, in samples incubated with streptavidin, the unbound nucleosomes were nearly completely digested by HhaI during the remodeling reaction (Figure 6, lanes 15–19, Nuc). In contrast, H2B S112C-MB nucleosomes bound by a single streptavidin were digested by HhaI during RSC remodeling, but at a slower rate compared to the unbound nucleosomes (compare 1SA and Nuc bands), while nucleosomes bound by two streptavidins were hardly digested at all (Figure 6, lanes 15–19, 2SA). Quantification of these data show that the single streptavidin-bound nucleosomes digest at a rate ∼2-fold slower than the unbound nucleosomes, while the double streptavidin-bound nucleosomes digest at a rate ∼20-fold slower than the unbound control (Supplementary Figure S5). These results are consistent with a near-complete inhibition of RSC-dependent Hha I site exposure when both protein faces are blocked by streptavidin, while the 2-fold reduction in remodeling of single SA-bound nucleosomes suggests complete inhibition of nucleosome remodeling for only one of two possible productive nucleosome-binding orientations by the RSC complex.

**Figure 7.**
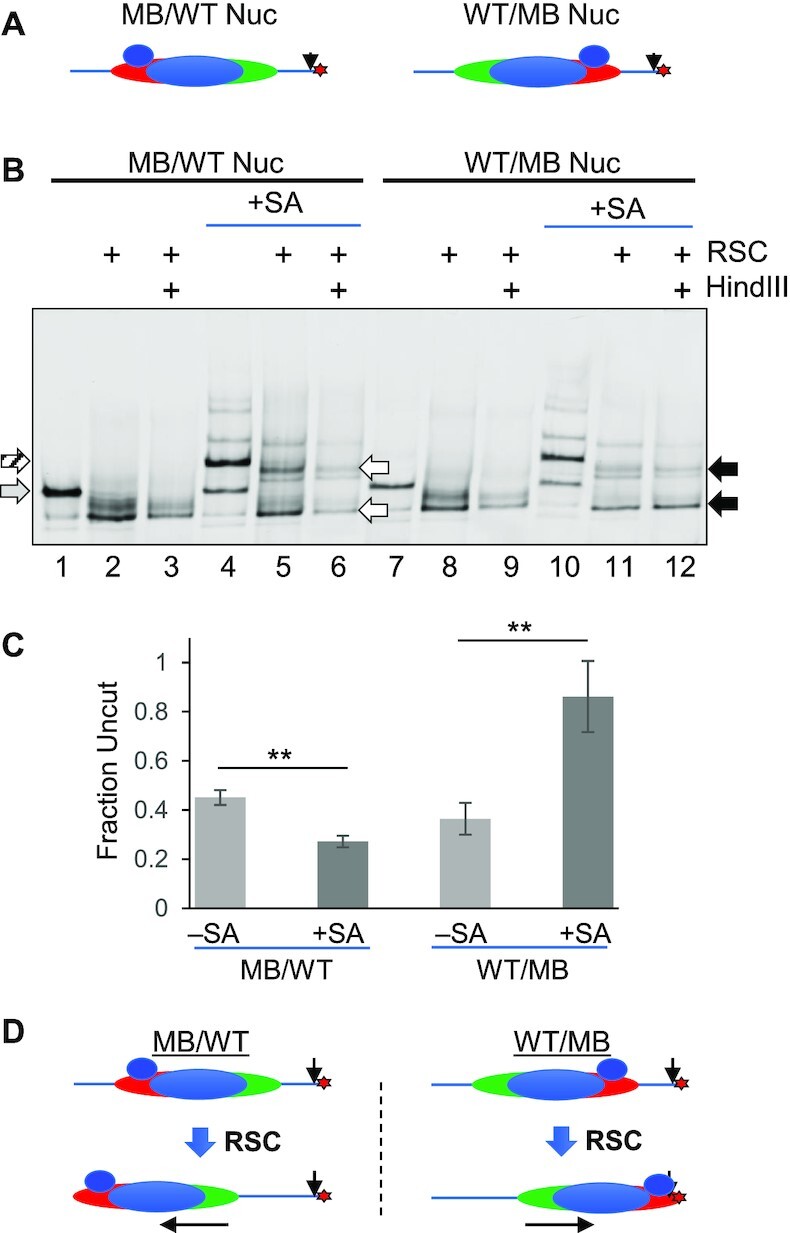
Asymmetric modification dictates direction of RSC-dependent nucleosome movement. (**A**) Asymmetric nucleosomes were reconstituted with biotin maleimide modified H2A/H2B S112C dimers (red) in either the upstream (MB/WT) or downstream (WT/MB) position (see also Supplementary Figure S5). WT H2A/H2B (green), H3/H4 tetramers (blue), Cy5 label (red star) and the Hind III site (arrow) are indicated. (**B**) Direction of mobilization of asymmetric nucleosomes by RSC is linked to nucleosome surface accessibility. Nucleosomes were incubated in the absence (lanes 1–3, and 7–9) or presence (lanes 4–6 and 10–12) of streptavidin (SA), then remodeled with RSC, as indicated, and the reaction stopped with excess plasmid DNA. Samples were either incubated without (lanes 2, 5, 8 and 11) or with Hind III and then loaded on the gel (lanes 3, 6, 9, and 12). Unbound and streptavidin-bound nucleosomes are indicated by the grey and striped arrows, left, respectively, while remodeled MB/WT and WT/MB nucleosomes are indicated by the white and black filled arrows, respectively. (**C**) Quantification of HindIII digestion indicates distinct nucleosome mobilization. Note two products of RSC remodeling are resolved on the gel (44), a slid nucleosome and a slid hexamer, in which the leading H2A/H2B dimer is lost (see lanes 2 and 8, and Supplementary Figure S7A and B). Therefore, remodeling of SA-bound nucleosomes generates a mobilized octamer bound by streptavidin and a mobilized hexamer in which the streptavidin-bound dimer is lost (see Supplementary Figure S7, C and D), and thus both species (indicated by the white and black arrows) were included in the quantification (*N* = 3). (**D**) Schematic of nucleosomes before and after RSC remodeling.

RSC remodeling of a nucleosome originally positioned at the center of a 601 DNA fragment results in mobilization to both ends of the fragment (38). Based on our observation that RSC remodeling exposes the central Hha I site in single-SA modified nucleosomes at a rate of about half of that for unmodified nucleosomes (Supplementary Figure S4), we hypothesized that RSC associates with nucleosomes bound by a single streptavidin in only one of two possible remodeling-productive orientations. We further hypothesized that each of the two possible orientations would correlate with nucleosome mobilization in a specific direction along the DNA, resulting in accumulation at a specific end of the DNA fragment. To test this idea, we generated asymmetrically modified nucleosomes in which the biotin-modified H2A/H2B S112C dimer was located either on the upstream or downstream end of the nucleosome, allowing binding of streptavidin to a defined side of the nucleosome relative to the DNA sequence (Supplementary Figures S6 and 7A) (30,31). We then asked whether RSC mobilization of asymmetric SA-H2A S112C nucleosomes exhibited a bias toward accumulation at a specific end of the DNA fragment by assessing the extent to which a Hind III site at the downstream end of the DNA fragment becomes protected from cleavage upon RSC remodeling (Figure 7A). Asymmetric nucleosomes containing a MB-modified dimer in the upstream position and a WT dimer in the downstream position (MB/WT) or visa-versa (WT/MB) were both efficiently mobilized by RSC in the absence of SA, resulting in partial protection (∼40%) of the Hind III site (Figure 7B, lanes 1–3, and 7–9, and Figure 7C, light grey bars). Indeed, the extent of protection implies nucleosomes were mobilized by RSC roughly equally to each end of the DNA fragment. Addition of streptavidin shifted the majority of both MB/WT and WT/MB nucleosomes on the native gel to a position corresponding to one streptavidin bound per nucleosome, as expected (Figure 7B, lanes 4 and 10). While both nucleosomes exhibited mobilization upon RSC remodeling, mobilized SA-bound MB/WT nucleosome products were nearly completely digested by Hind III, while mobilized SA-bound WT/MB nucleosome products were largely resistant to Hind III digestion (Figure 7B, compare lanes 5 to 6, and 11 to 12, white and black arrows, respectively). Indeed, quantification showed that the fraction of WT/MB and MB/WT nucleosomes protected from Hind III digestion was ∼20% and ∼80%, respectively (Figure 7C, dark grey bars), indicating a striking bias in the direction of nucleosome mobilization. Moreover, SA-binding resulted in a significant *decrease* in the fraction of MB/WT nucleosomes protected from Hind III, while the fraction of WT/MB nucleosomes protected from Hind III cleavage significantly *increased* (Figure 7C, compare green and grey bars). We conclude that asymmetric binding of streptavidin restricted movement of MB/WT nucleosomes only to the upstream end of the DNA fragment, while, in contrast, restricting movement of WT/MB nucleosomes to the downstream end of the fragment (Figure 7D, and Supplementary Figure S8).

## DISCUSSION

We find that selectively restricting accessibility to the each of the protein faces in the nucleosome can define the direction of nucleosome mobilization driven by the RSC ATP-dependent chromatin remodeling complex. By preparing asymmetrically modified nucleosomes, we find mobilization only toward the upstream end of the DNA fragment upon blocking the upstream nucleosome face, while blocking the downstream-oriented face results in exclusively downstream movement of the nucleosome. These results have direct consequence for understanding RSC movement of nucleosomes in the vicinity of promoters, as RSC is thought to mobilize nucleosomes out of the nucleosome-depleted region, allowing binding of general transcription factors (12).

Recent cryoEM structures illustrate that the RSC complex interacts with several nucleosome surfaces, including both nucleosome faces, via three main multi-subunit lobes (23–25). The motor domain, comprised of the ATPase domain of Sth1, interacts with DNA SHL2 to pull DNA in from the proximal (leading) edge of the nucleosome (Figure 1). The N-terminus of Sth1 contains SnAc and bromo domains positioned to interact with the nearby proximal nucleosome face and/or acetylated core histone tail domains (25). A similar interaction has recently been reported for the SnAc/bromo region of the SMARCA4 subunit in the related PBAF-nucleosome complex (27). The nucleosome face distal to the motor module is contacted by the multi-subunit SRM module, comprised of the C-terminus of Sth1, and numerous subunits containing bromodomain and BAH domains, including Rsc1/2, Rsc4 and the N-terminus of Sth1, which are positioned to interact with acetylated histone tails, including H3 K14ac surrounding the distal face. Moreover, the Sfh1 subunit contains a basic α-helix, which interacts possibly with the H2A/H2B acidic patch on the distal face of the nucleosome (24,25).

Our data indicate that blocking both protein faces of the nucleosome with streptavidin eliminated remodeling-dependent exposure of the Hha I site at the nucleosome dyad and nucleosome mobilization. Thus, such a similarly modified nucleosome via binding of ancillary factors would be impervious to RSC remodeling *in vivo*. Moreover, we find that blocking one protein face of the nucleosome reduced the rate of HhaI site exposure to about half that of control, suggesting loss of one of two productive binding orientations. Indeed, using asymmetrically modified nucleosomes we found efficient remodeling in one direction, consistent with one allowed RSC binding orientation. Based on the known direction of DNA movement dictated by the Sth1 ATPase motor domain (5), our results indicate interactions of the SRM domain are essential for nucleosome mobilization (Figure 1). In contrast, we find that occlusion of the protein face proximal to the Sth1 motor module is compatible with efficient remodeling and nucleosome mobilization toward the proximal nucleosome face (Figure 1). These results indicate that interactions between the SnAc region of Sth1 and/or the Sth1 bromo domain with the proximal nucleosome protein surface are not critical for RSC nucleosome mobilization, while extensive interactions between RSC subunits comprising the SRM module and interacting the proximal face are essential for nucleosome mobilization. These results also indicate a potential critical difference between the SWI/SNF and RSC complexes (26).

Prior work found that RSC complex containing a deletion of the arginine-rich CTT domain within Sfh1 exhibits nucleosome sliding but does reduce nucleosome eviction from plasmids (23). However, while the Sfh1 CTT likely represents the majority of interactions with the nucleosome from the NB lobe of the SRM domain, as described above, many components of the 3-lobed SRM likely contribute to binding the distal nucleosome face (23–25). Moreover, attachment of streptavidin represents addition of a 66 kDa mass to the nucleosome surface, similar to that provided by binding of an ancillary protein factor to a specific set of epigenetic modifications. Attachment of a large mass to the nucleosome protein surface, or multiple smaller modifications may be required to abrogate a sufficient number of SRM interactions to affect RSC binding.

Our findings indicate a mechanism for enforcing unidirectional movement by RSC *in viv*o, as has been found for mobilizing nucleosomes away from the nucleosome-depleted regions upstream of active genes (12). For example, post-translational modifications such as ubiquitination, or binding of ancillary factors to the nucleosome face, or any occlusion that would hinder the relatively large nucleosome-binding SRM domain access will be accommodated on the nucleosome proximal face and thus only allow nucleosome mobilization in the direction of the occluded face (Figure 1 B and C). Interestingly, it has been reported that ubiquitination of H2B at residue 120 within the acidic patch diminishes RSC function *in vivo* (39), and that several arginines in the Sfh1 helix are mutated in cancers. ISWI remodelers are dependent on the H2A/H2B acidic patch on the nucleosome surface (40), and posttranslational modifications in the vicinity of the patch can regulate function of several remodeling complexes, including SWI/SNF (41). In addition, a recent report demonstrates that the human BAF SS18-SSX (synovial sarcoma X breakpoint) protein, a fusion protein that is a driver of synovial sarcoma, directs binding of the SWI/SNF complex to H2A K119-ubiquitinated nucleosomes in heterochromatin regions by tethering the SRM to the distal acidic patch of nucleosomes to de-repress gene expression (42). In this case, directed movement to de-repress of gene expression could instigated by directional ubiquitination of the nucleosome. Regardless, in combination, these results suggest uni-directional mobilization of nucleosomes may be a primary activity of nucleosome remodeling complexes.

We also observed that blocking the DNA surfaces in the ‘back’ of the nucleosome, near SHL ±4.5 resulted in inhibition of nucleosome mobilization but did not hinder RSC-dependent exposure of a Hha I site at the nucleosome dyad. These results indicate an uncoupling of mobilization and internal DNA site exposure during remodeling, as previously observed for RSC (35,36). Moreover, these assays highlight an important difference between streptavidin binding to the two sites explored in our work. H2A N-tail modification does not block productive enzyme binding, thereby allowing DNA site exposure, but does block one outcome of the reaction (mobilization). In contrast, modification to the nucleosome faces blocks productive enzyme binding in an orientation-dependent manner, eliminating both DNA site exposure and nucleosome mobilization.

Moreover, inspection of models for the RSC-nucleosome complex suggests, no obvious steric interference between streptavidin attached at SHL ±4.5 and any lobe or domain of the RSC complex (23–25). However, we previously showed that crosslinking the two H2A tails together results in a diminution of RSC-dependent nucleosome mobilization, but not a complete block. Its therefore possible that the RSC motor domain ‘pulls’ DNA into the nucleosome from the proximal end in a manner that does not require significant liberation from the nucleosome surface (such as a loop), but DNA deformations accumulate downstream of the motor domain/SHL 2 contact point which lead to accessibility of the HhaI site at the dyad. Moreover, our data suggest that dissipation of internal DNA loops to the distal region of the nucleosome core may be hindered by the streptavidin attachment point, thereby resulting in significantly reduced nucleosome mobilization. Note that since the SA modification lies along on the nucleosome symmetry axis, there is no distinction between upstream/downstream orientations.

We also note that remodeling of nucleosomes generates both slid octamers and hexamers (Figure 7B, lane 2; Supplementary Figure S7), in which the leading H2A/H2B dimer is lost due to loss of histone-DNA contacts as the histones are mobilized beyond the end of the DNA fragment, as has been observed previously (21,43,44). Moreover, our observation that mobilization of asymmetrically streptavidin-bound nucleosomes generates hexamers in which the modified dimer is lost (Figure 7B and Supplementary Figure S7) nicely aligns with our data and interpretation. The SA modification forces the modified dimer to be the leading face of the nucleosome during movement, thus only the SA-bound dimer is pushed off the end of the fragment and is lost from the nucleosome.

## DATA AVAILABILITY

All data is available upon request.

## Supplementary Material

gkac790_Supplemental_File
