# Supplementary material for: Histone protein surface accessibility dictates direction of RSC-dependent nucleosome mobilization
Source: Nucleic Acids Res. 2022 Sep 26;50(18):10376–84. doi: 10.1093/nar/gkac790 (PMC9561379; doi:10.1093/nar/gkac790)
Supplement: gkac790_Supplemental_File [file gkac790_supplemental_file.pdf]

Histone protein surface accessibility dictates direction of RSC-dependent nucleosome mobilization

Supplemental Figures

Javeed Bhat†, Angela J. Balliano† and Jeffrey J. Hayes*

Department of Biochemistry and Biophysics, University of Rochester Medical Center, Rochester, New York, 14642

†These authors contributed equally to this work

*Corresponding author: Jeffrey_Hayes@urmc.rochester.edu

PRIMERS
TC218F: CGACTGGCACCGGCAAGG
TC218R: CATCCCTTATGTGATGGAC
233Cy5HindIIIR:
CATc[Cy5]CCTTAAGCTTATGTGATGGACCCTATACG

218 bp fragment generated by TC218F and TC218R:
CGACTGGCACCGGCAAGGTCGCTGTTCAATACATGCACAGGATGTATATATCTGACACGTGCC
TGGAGACTAGGGAGTAATCCCCTTGGCGGTTAAAACGCGGGGGACAgCGCGTACGTGCGTTT
AAGCGGTGCTAGAGCTGTCTACGACCAATTGAGCGGCCTCGGCACCGGGATTCTCCAGGGC
GGCCGCGTATAGGGTCCATCACATAAGGGATG **218 bp**

224 bp fragment generated by TC218F and 233Cy5HindIIIR:
CGACTGGCACCGGCAAGGTCGCTGTTCAATACATGCACAGGATGTATATATCTGACACGTGCC
TGGAGACTAGGGAGTAATCCCCTTGGCGGTTAAAACGCGGGGGACAgCGCGTACGTGCGTTT
AAGCGGTGCTAGAGCTGTCTACGACCAATTGAGCGGCCTCGGCACCGGGATTCTCCAGGGC
GGCCGCGTATAGGGTCCATCACATAAG<u>CTTAAG</u>G**G**ATG **224 bp**

Table S1. The small case g denotes the dyad base position, the Hind III site is underlined, and the Cy5 is attached to the C opposite the bolded G in the lower sequence. Note that the 'strong' H2A/H2B dimer binding side is downstream of the dyad position in this sequence (1) (see also Fig. S6)

Fig S1

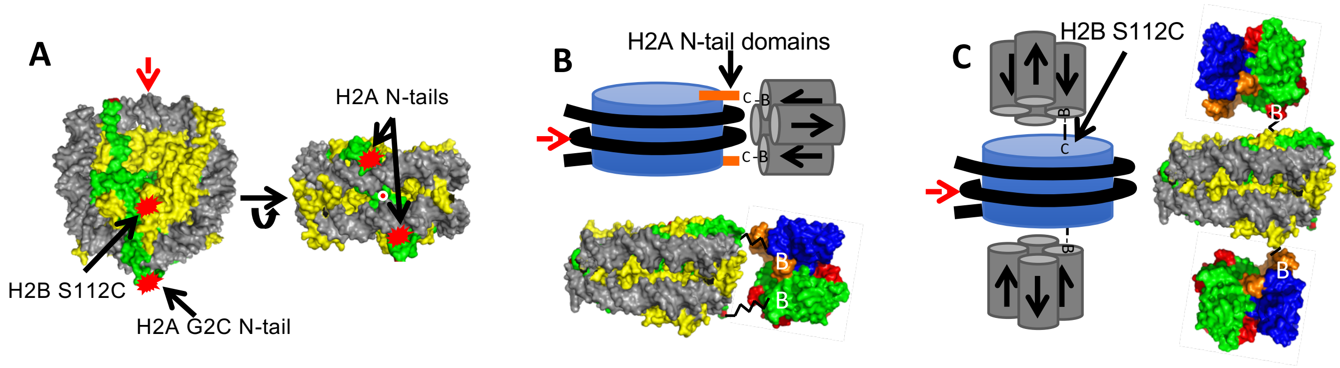

Fig. S1. Position of streptavidin attachment on the nucleosome surface. A. Sites of biotin/streptavidin attachment in the nucleosome. Cysteines were placed in the N-terminal tail domain of H2A (H2A G2C) and on the protein face of the nucleosome (H2B S112C), indicated by red starbursts. H2A is shown in green, other core histones are yellow, and DNA is grey. The nucleosome dyad is indicated by the red arrow and red dot. Views shown are down the superhelical axis (left) and along the dyad axis (right). Model of the nucleosome based on PDB 1KX5 (2). B. Model of streptavidin attachment to H2B S112C-MB nucleosomes. A cartoon model (top) and molecular model (bottom) are shown. View is 90° rotated from that shown in A, right. Top. Arrows indicate the orientation of monomers within the streptavidin tetramer, with biotin (B) near the two confacial binding sites. Bottom. Molecular model of the nucleosome-streptavidin complex. PEG2 tether (black lines) are shown, with each streptavidin monomer colored green, blue, red and orange. Streptavidin model adapted from PDB 1SWD (3). C. Models of H2A S112C nucleosome-streptavidin complex. Shown are a cartoon and molecular model of the nucleosome and streptavidin, as in B.

Fig. S2

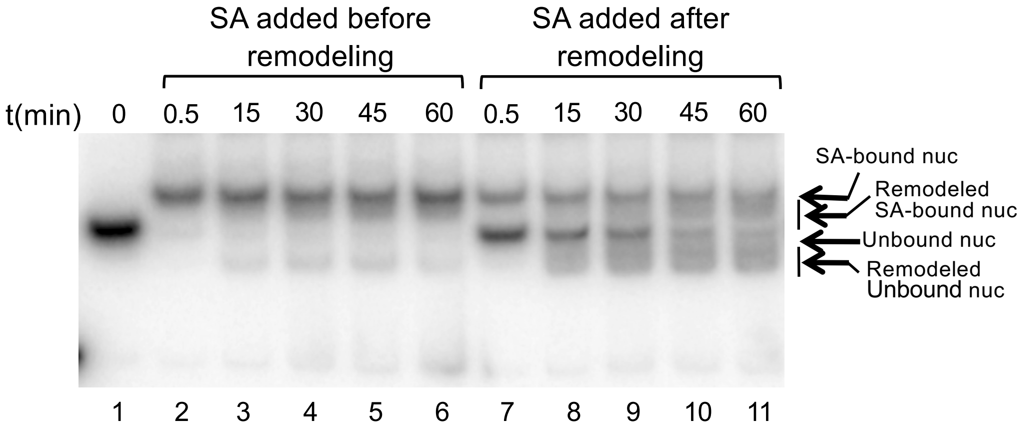

Fig. S2. Streptavidin binding to H2A G2C-MB nucleosomes does not mask RSC-dependent mobilization on gels. Streptavidin was incubated with H2A G2C-MB nucleosomes either prior remodeling by RSC (lanes 2-6) or after remodeling (lanes 7-11) to reveal native gel motilities of bona fide RSC-remodeled SA-bound nucleosome species. Time points were taken immediately after the addition of RSC (0.5 min), and at 15, 30, 45, and 60 minutes and the remodeling stopped by addition of 200 ng plasmid DNA. Samples were analyzed on 6% native PAGE gels.

Fig. S3

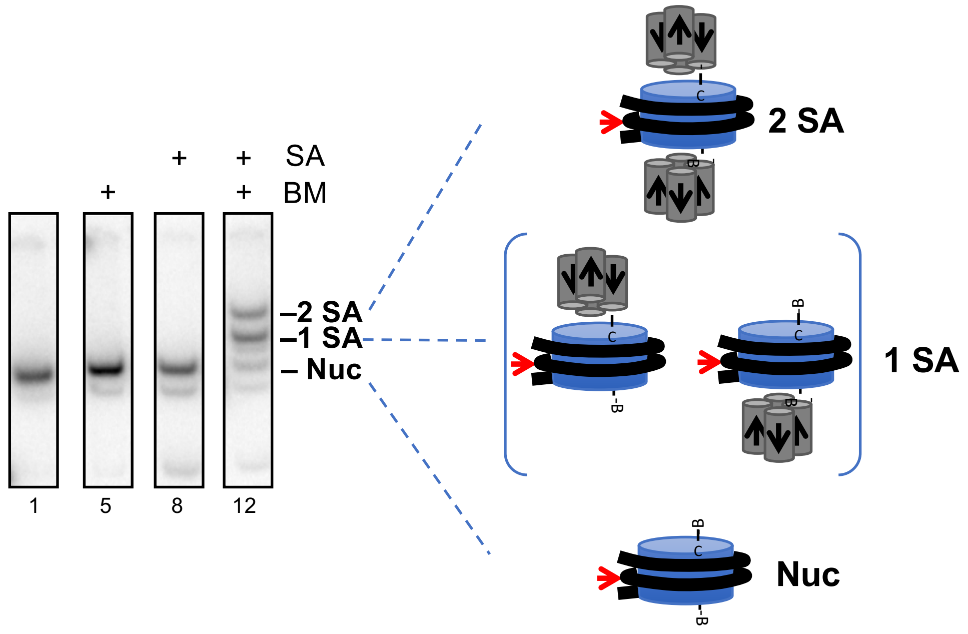

Fig. S3. Binding of SA to H2A/H2B S112C-MB nucleosomes yields a distribution of species. Selected lanes from Fig. 6 shown as indicated, for reference, along with corresponding species.

Fig. S4

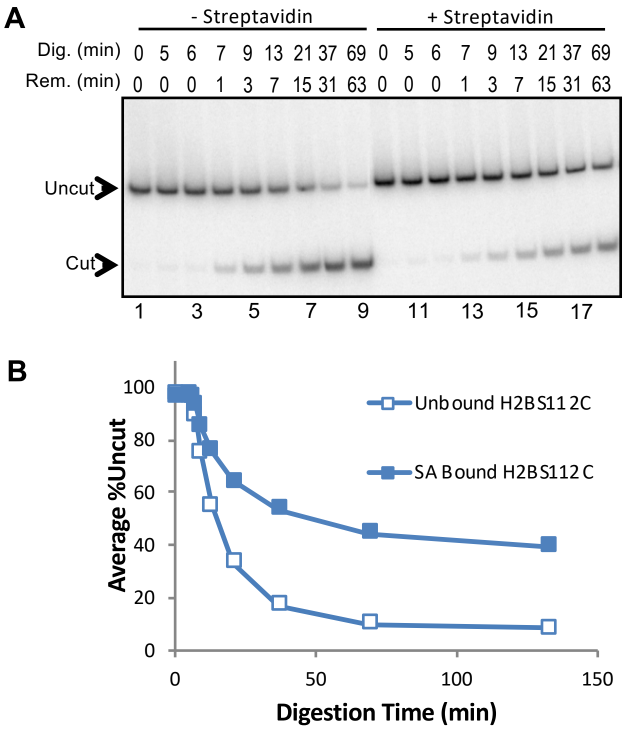

Fig. S4. Restriction enzyme accessibility assay with SA-bound H2B S112C-BM nucleosomes. Nucleosomes were digested with 5U HhaI before addition of RSC at the 6 min time point. The reaction was allowed to proceed and additional time points were taken at 7, 9, 13, 21, 37, 70, and 130 min. Reactions were stopped with SDS loading dye and samples were loaded onto a 6% PAGE containing 0.04% SDS which was run for 2.5 h at 120 V. A. Gel of the samples taken at indicated time points from digests of nucleosomes incubated in the presence (open squares) or absence (filled squares) of streptavidin. B. The percent of uncut DNA was quantitated from the gel and plotted vs. HhaI digestion time for SA bound (filled squares) and unbound nucleosomes (open squares). Three repeats of the experiment were performed, a representative one is shown.

Fig. S5.

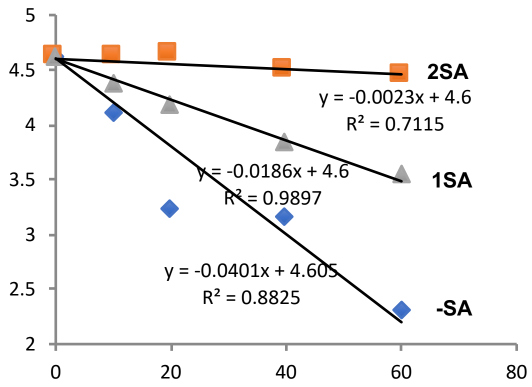

	Rate	Relative Rate
-SA	0.040	1.0
1SA	0.019	0.47
2SA	0.0023	0.058

Fig. S5. Rate of restriction enzyme digestion during RSC remodeling of streptavidin-bound surface mutants. The rates of Hha I digestion for H2A/H2B S112C-MB nucleosomes bound by 0, 1, or 2 streptavidins were determined by quantification of the corresponding bands found in Fig. 7, Lanes 15-19. Data was plotted and fitted to a linear regression (- SA, blue diamonds; 1SA, green triangles; 2SA, red squares). Parameters from linear fits are shown. The rates and relative rates are listed in Table 1.

Fig S6

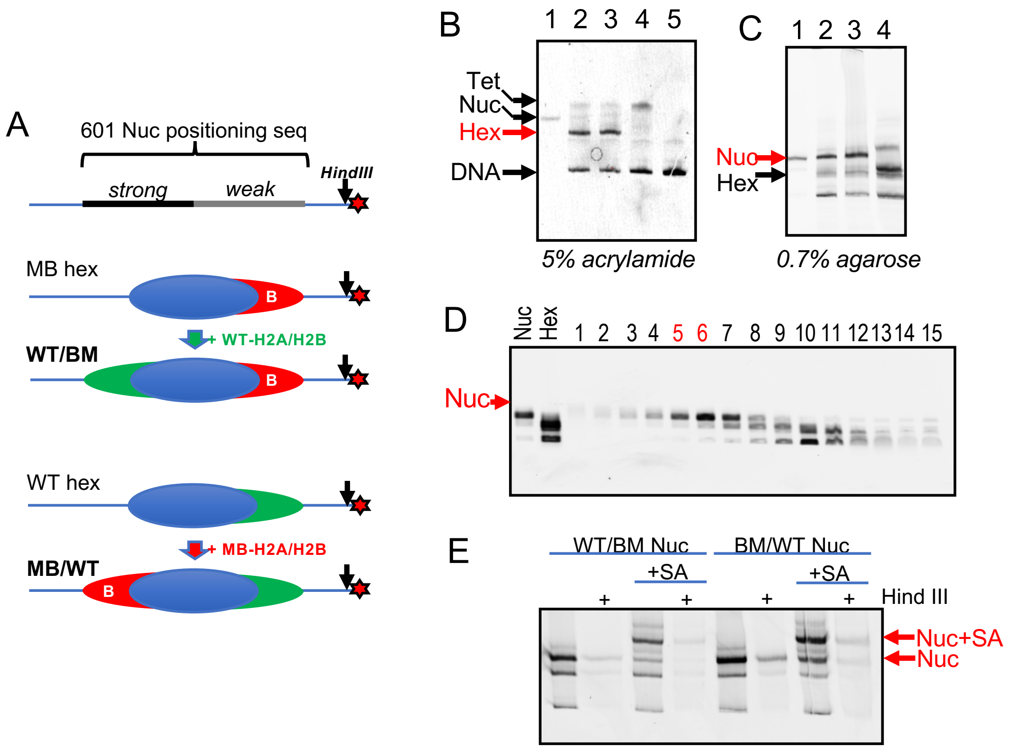

Fig. S6. Preparation of asymmetric nucleosomes. A. Scheme of preparation of WT- and BM-Hexamers containing either a WT H2A/H2B dimer (green) or H2A/H2B S112C-MB dimer (red), and corresponding asymmetric nucleosomes. B. Hexamer preparations. Lane 1, Nucleosome control; Lane 2, WT hexamer; Lane 3 BM Hexamer; Lane 4, H3/H4 tetramer-DNA complex, Lane 5, Cy5-DNA. C. Hexamer complexes shown in B were converted to asymmetric octamers as described in the Methods. Lane 1 WT nucleosome control; Lanes 2 and 3, Converted WT-BM and BM-WT nucleosomes, respectively; Lane 4 WT-hexamer control. D. Gradient purification of asymmetric nucleosomes. Direction of sedimentation is right to left. WT nucleosomes and hexamers are shown as controls. Fractions in lanes 5 and 6 were pooled for experiments. E. Hind III site is accessible in reconstituted nucleosomes.

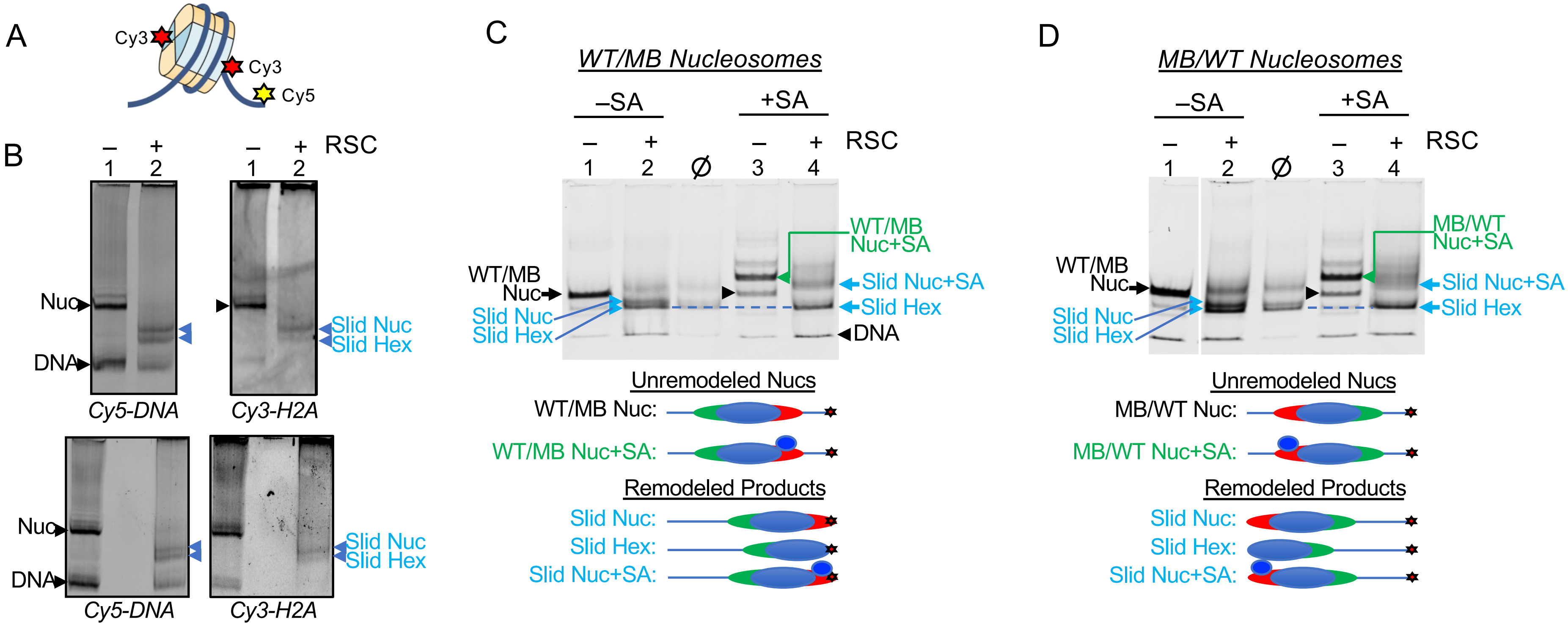

Fig. S7. RSC remodeling generates slid nucleosomes and hexamer-DNA complexes lacking one H2A/H2B dimer. A. Nucleosomes were generated in which the DNA was end-labeled with Cy5 and the two H2A/H2B dimers were labeled with Cy3 (H2A S128C). B. Remodeling generates two nucleoprotein species, one of which is deficient in H2A/H2B dimers. Products of nucleosome remodeling (+RSC) were separated on native PAGE gels and and Cy5 and Cy3 fluorograms obtained. The unremodeled nucleosomes (Nuc) and naked DNA are indicated (black arrowheads), as is the two "Slid" products of remodeling typically observed in our experiments. Two independent experiments were performed and are shown. The "Slid" bands were quantified by densitometry, Cy3 band normalized to the relative amount of each Cy5 band, and then the ratio of the lower to upper band determined for each replicate. The lower band was found to contain 52.3% of the H2A/H2B content of the upper band (N=2, as shown, 56.2%, 48.5%), with the upper band containing an equivalent ratio as the unremodeled nucleosomes. These data identify the lower band as a hexamer lacking an H2A/H2B dimer, as previously reported (4). C and D. Remodeling of asymmetric streptavidin (SA)-bound nucleosomes generates hexamers in which the SA-H2A/H2B S112C-MB dimer is lost. Note that remodeling of SA-bound nucleosomes (WT/MB or MB/WT) generates only the *lower* band corresponding to the hexamer (Slid Hex); slid nucleosomes (Slid Nuc+SA) run at a higher position on the gel.

Fig S8

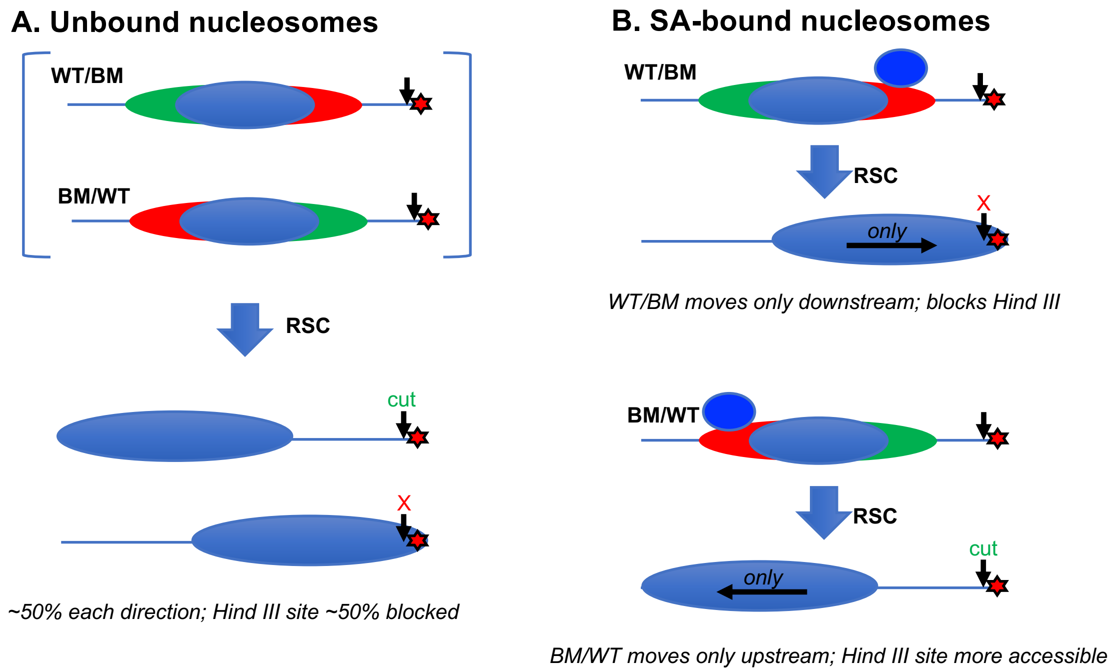

Fig. S8. Scheme of effect of asymmetric SA binding on direction of nucleosome mobilization. A. WT/BM and BM/WT nucleosomes in the absence of streptavidin (SA) are mobilized approximately equally to the upstream and downstream ends of the DNA fragment. The Hind III site (black arrow) is ~50% blocked. B, Asymmetric SA binding (blue oval) defines direction of nucleosome mobilization by RSC. Top, the SA-bound WT/BM nucleosome is mobilized only to the downstream end of the DNA fragment, blocking the Hind III site, as only one RSC binding mode is allowed Mobilization to the upstream end is blocked by SA. Bottom, The SA-bound BM/WT nucleosome is mobilized to only the upstream end of the DNA fragment, as asymmetric SA blocks the RSC binding orientation directing movement downstream. Hind III accessibility is maximal in these samples. Note mobilization generates both slid nucleosomes and hexamers, which one H2A/H2B dimer is evicted due to loss of histone-DNA contacts (See Fig. S7).

SI References

1. Levendosky, R.F., Sabantsev, A., Deindl, S. and Bowman, G.D. (2016) The Chd1 chromatin remodeler shifts hexasomes unidirectionally. eLife, 5:e21356. 10.7554/eLife.21356.

2. Davey, C.A., Sargent, D.F., Luger, K., Maeder, A.W. and Richmond, T.J. (2002) Solvent mediated interactions in the structure of the nucleosome core particle at 1.9 a resolution. J. Mol. Biol., 319, 1097-1113.

3. Freitag, S., Le Trong, I., Klumb, L., Stayton, P.S. and Stenkamp, R.E. (1997) Structural studies of the streptavidin binding loop. Protein Sci, 6, 1157-1166.

4. Bruno, M., Flaus, A., Stockdale, C., Rencurel, C., Ferreira, H. and Owen-Hughes, T. (2003) Histone H2A/H2B dimer exchange by ATP-dependent chromatin remodeling activities. Mol Cell, 12, 1599-1606.
